# Examination of Performance Levels of Wheelchair Basketball Players Playing in Different Leagues

**DOI:** 10.3390/sports6010018

**Published:** 2018-02-25

**Authors:** Mehmet Fatih Yüksel, Tarık Sevindi

**Affiliations:** 1 Faculty of Education, Necmettin Erbakan University, Konya 42090, Turkey; 2 Faculty of Sports Science, Aksaray University, Aksaray 68100, Turkey; tariksevindi@gmail.com

**Keywords:** wheelchair basketball, disabled, physical features

## Abstract

This research was conducted to examine the anthropometric and biometric features of the elite wheelchair basketball players in different league levels, and to evaluate them with regards to field tests particular to wheelchair basketball. A sample of 21 male players volunteered to participate in the research with similar classification points, 12 of whom were from Turkey Wheelchair Basketball First League and 9 of whom were from the Second League. Anthropometric measurements, biometric features of the players and their skill test scores particular to wheelchair basketball were detected. The anthropometric measurements were taken over dominant extremity. SPSS 21.0 program was used in the analysis of the data, and minimum, maximum, arithmetic mean, and standard deviation values were determined. Intergroup differences were determined with Mann–Whitney U analysis. Significance level was admitted as *p* < 0.05. As a conclusion, it was determined that wheelchair basketball players had similar anthropometric features in the First and Second League levels, and that there was no difference based on the league level they were playing, and moreover, that bio-motor features and skills particular to wheelchair basketball were decisive on the levels of the leagues the players were taking part.

## 1. Introduction

There are more than 1 billion people with disability globally, that is about 15% of the world’s population or one in seven people. Of this number, between 110 million and 190 million adults experience significant difficulties in functioning [1,2]. Considering the World Health Organization (WHO) and United Nations (UN) reports, exercise and sport gains much more importance for the individuals who have limited physical activity due to physical disabilities. Currently, it is observed that disabled sportsmen and sportswomen show great paces of improvement and gain big prizes, since disabled sport branches are sought after [3,4,5,6,7]. There are numerous adapted sport branches for individuals with disabilities and wheelchair basketball is one of the most popular ones.

A fast and exciting sport, wheelchair basketball has the same standards with able-bodied basketball [8]. It is characterized with high intensity activities such as spinning the wheel, rebounding, passing, shooting, fast and sudden moves to back and front, and short sprints [9,10,11]. Contrary to many sport branches performed by the disabled, wheelchair basketball is a team game and it is carried out with the participation of disabled individuals with different functional levels [8]. The functional classification model was formally adopted by International Wheelchair Basketball Federation [12].

When the literature is examined, it is observed that there are useful and helpful studies conducted on wheelchair basketball players. Previous studies have observed that there are studies examining the upper extremity functional levels in individuals mobilized with wheelchair, reporting that upper extremity muscle strength, speed, and endurance have an important role [10,13]. There are other research studies examining physical fitness of wheelchair basketball players [14] or evaluating technical skills [15]. Some other research studies scrutinized the difference between functional classification levels and disability types [16], searched the core stabilization findings [17], and observed the effect of the wheelchair playing periods on physical fitness and life quality [18]. However, with regards to the literature scanned, it is observed that, in many studies [10,14,15,16,18], the wheelchair basketball players are evaluated as players with functionally lower upper-body control (1 vs. 2.5) and higher upper-body control (3 vs. 4.5) according to the disability points determined by the International Wheelchair Basketball Federation (IWBF). From this point of view, the wheelchair basketball players with similar classification points were not evaluated with regards to anthropometric, biometric, and technical skills in different league levels, and possible differences between the leagues were not examined, which motivated this research to be carried out.

This research was conducted to examine the anthropometric and biometric features of the elite wheelchair basketball players in different league levels, and to evaluate with regards to field tests particular to wheelchair basketball.

## 2. Materials and Methods

### 2.1. Participants

The subjects of this research were chosen among the volunteer players. 21 male players participated in the research, 12 of whom were from the 68 Aksaray Municipality Sports Club in Turkey Wheelchair Basketball First League, and 9 of whom were from the Second League Konya Disabled Power Sports Club. Necessary permissions for conducting tests and measurements were taken from authorities of the 68 Aksaray Municipality and Konya Disabled Power Sports Club. All the volunteers participating in the research signed the informed consent (volunteer) form and filled personal information form. The study was conducted in accordance with the Declaration of Helsinki, and the protocol was approved by the Necmettin Erbakan University, Meram Medical Faculty, Ethics Committee of Non-Pharmaceuticals and Non-Medical Device Research with the decision dated 1 December 2017 and numbered 2017/1108.

### 2.2. Place and Time of Measurement

All of the measurements and tests of the basketball players composing the experiment group were carried out in Aksaray Hasandağı Sports Hall and Konya Martyr Rıdvan Adam Sports Hall. They were recorded in the interval period of the league during the 2017/2018 season. Disability classification of the players was determined before the tests and measurements.

### 2.3. Disability Condition

When the participant players from the First League were examined; it was determined that the volunteered players composed of 2 (*n* = 2) congenital, and 10 (*n* = 10) acquired disabled individuals; and that 8 of them were poliomyelitis (*n* = 8), 1 person was lower-extremity unilaterally amputated (*n* = 1), 2 people had paraplegia (*n* = 2), 1 person had with Guillain Barre syndrome (*n* = 1); 2 individuals had a prosthesis (*n* = 2), 4 individuals had crutches (*n* = 4) and act independently (*n* = 2), and 4 individuals were using wheelchairs (*n* = 4). When the participant players from the Second League were examined, it was determined that the volunteered players composed of 1 (*n* = 1) congenital, and 8 (*n* = 8) acquired disabled individuals; and that 4 players were poliomyelitis (*n* = 4), 3 individuals were lower-extremity unilaterally amputated (*n* = 3), 2 of them were paraplegic (*n* = 2); 3 individuals had prosthesis (*n* = 3), 3 of them had crutches (*n* = 3), and 3 players were using wheelchairs (*n* = 3).

### 2.4. Procedures

Some limitations were applied considering the physical disability condition during the tests and measurements. Anthropometric measurements were conducted on dominant extremities. Modified sit-up, modified abdominal endurance, and modified push-up values of only 10 players were determined due to lack of consent for participation of 2 sportsmen among the First League wheelchair basketball players. The tests and protocols that were conducted to determine the physical features of the players are explained below.

Within the context of the research, the measurements were taken with regards to numerous parameters. Stature, body weight, and body mass index (BMI) were determined according to the method reported by Zorba and Saygın [19]. The measurement method reported by Chen et al. [20], Easterby et al. [21], Otman et al. [22] were used in taking the measurements of upper-body length, upper extremity length, arm length, forearm length, hand length, arm circumference, and forearm circumference. A modified functional reaching test, which was reported by Özünlü and Ergun [23], was used in order to evaluate upper body balance. A modified sit-up test was implemented according to the description of Sahlberg et al. [24] and Tomchuk [25]. Modified abdominal endurance and modified push-up tests were applied in the context of the explanation of Ergun and Baltacı [26]. For determining the hand grasping power, the method reported by Günay et al. [27] was based on. For the plate tapping test, the method recommended by Adam [28] was applied. Shoulder flexibility was measured via Back Scratch test and the method used by Dewhurst and Bampouras [29] was implemented in the measurement. For pass for distance and lay up tests, the criteria determined by Zacharakis et al. [15] were grounded on. As for the 20-m speed test and zone shot test, the method that was used by Vanlandewijck et al. [30] was chosen. The recommendations of Molik et al. [16] were implemented for the slalom without ball test and slalom with ball test. Pass for accuracy test and 6-min endurance race test were recorded according to the methodology of Ergun et al. [18].

### 2.5. Statistical Analysis

SPSS 21.0 program was used in the analysis of the data obtained in the study, and minimum, maximum, arithmetic mean, and standard deviation values were determined. Inter-group differences were determined with Mann–Whitney U analysis. Significance level was admitted as *p* < 0.05.

## 3. Results

When the Table 1 is examined, according to the Mann–Whitney U analysis conducted to determine whether there was a significant difference between the average values of the First and Second League wheelchair players, it was determined that there were statistically significant difference (*p* < 0.05) on training age (U = 24.000) parameters.

When the Table 2 is examined, according to the Mann–Whitney U analysis conducted to determine whether there was a significant difference between the average values of the First and Second League wheelchair players, it was determined that there were statistically significant difference (*p* < 0.05) on modified sit-up (U = 17.000), modified abdominal endurance (U = 12.000), dominant hand plate tapping (23.000), left shoulder flexibility (U = 22.500), pass for distance (U = 25.500), 20-m speed (U = 25.500), slalom without the ball (U = 7.000), slalom with the ball (U = 19.000), zone shot (U = 19.500), pass for accuracy (U = 21.500), and 6-min endurance race test (U = 16.000) parameters, while statistically there was no significant difference (*p* > 0.05) on the other parameters.

## 4. Discussion

This research examined the anthropometric and biometric features of the elite wheelchair basketball players in different league levels, and to evaluate with regards to field tests particular to wheelchair basketball. The points that the players received from the functional classification system is important in wheelchair basketball, as is the case with all sport branches in which disabled sportsmen and sportswomen participate. The IWBF classification score is from 1 to 4.5. According to the classification scores, the players are given appropriate points. It is classified as 4.5 points for the players with minimal disabilities. The total score of five players playing in the competition must not exceed 14. This point differences are important with regards to checking the upper-body and extremities and determining the functional levels of the players. In this research, it was observed that the classification points of the First and Second League wheelchair basketball players were close (2.75 and 2.83) to each other, and that there was statistically no significant difference. In this respect, despite the differences between the functional levels of the players, considering the team averages, it is considered important to determine whether the inter-league differences stemmed from either anthropometric/biometric features or with regards to field tests. Therefore, it is aimed to contribute the findings of this study to the literature, and moreover, to render them as a source for trainers/sports scientists and other shareholders.

It is observed that wheelchair basketball players of the First League were older and taller, had lower body weights and lower body mass indexes compared to the Second League basketball players; however, these differences were statistically not significant. Besides, it was determined that there were statistically significant differences in favor of the First League players in training age, modified sit-up, modified abdominal endurance, dominant hand plate tapping, left shoulder flexibility, pass for distance, 20-m speed, slalom without the ball, slalom with the ball, zone shot, pass for accuracy and 6-min endurance race test parameters, and moreover, better test scores were obtained in all other parameters.

It is stated that individuals dealing with sports are more independent in terms of mobility in the daily life and have higher life quality compared to individuals who do not deal with sports, and that regular exercises of a disabled individual were important regarding the physical fitness [31,32,33]. Ergun et al. [18] reported that the playing age of the basketball players has an important influence on the physical fitness and there is a positive relation between basketball playing age and some skill tests. We evaluate the motive behind the fact that the First League wheelchair basketball players had better scores on many parameters in this research than the Second League players, is that the training ages (11.66 vs. 4.77) of the First and Second League players have statistically significant difference. This determination is supported by Hutzler et al. [31], Wilhite and Shank [32], Yazıcıoğlu et al. [33], Ergun et al. [18], and Cömert et al. [10] as well.

With regards to anthropometric measurement results, it was determined that the average values of the wheelchair basketball players in the First League were higher compared to the Second League players; however, this difference was statistically not significant. On the other hand, statistically significant differences were located in favor of the First League players on bio-motoric test results such as abdominal power and endurance, shoulder flexibility, upper-body balance, arm move speed, and hand grasping power. This situation can be attributed to that the players in the Second League allocate less time for abdominal power and endurance, arm move speed, and flexibility exercises in their training programs. Abdominal power and endurance have positive effects on upper-body stabilization and balance, particularly for wheelchair basketball sport branch. Therefore, another indicator for this situation can be explained by that they firstly met a left shoulder flexibility test (it was understood in the interviews with the Second League players that they did not conduct core exercises and moreover they only exercised shoulder flexibility moves on the right shoulder). Additionally it is reported that the motoric feature differences diminish as the professional level players mature physiologically and anatomically [34] and it is obvious that the findings of this study have parallels with this principle.

With regards to field tests conducted within the study (pass for distance, 20-m speed, slalom without the ball, slalom with the ball, zone shot, pass for accuracy, 6-min endurance race), it was determined that there were statistically significant differences in favor of the wheelchair basketball players in the First League. We evaluate that the main reason behind these differences is the differences between the training ages. It was also verified by the information about the weekly training program learned from the players. Van der Woude et al. [13] and Akınoğlu et al. [17] reported that muscle power of upper extremity and core region, and cardiovascular endurance are quite important for the individuals mobilized with wheelchair. In another study, it was similarly stated that parameters of the players such as upper extremity muscle power, endurance, and speed gained vital importance for both performing activities (shooting, pass, and rebound) particular to sports and controlling the wheelchair [10]. Although we agree with the reported literature data [10,13,17], we consider that most of the field tests particular to wheelchair basketball depend on wheelchair using ability. In this respect, it is a necessity to regularly include exercises improving the wheelchair using ability in the trainings.

Within the accessible literature findings [14,15,16,18] regarding field tests particular to wheelchair basketball and considering the classification points, it was determined that the test scores of many parameters conform with the players in the 1st League and that they have better average values than the Second League players. We are of the opinion that these differences were originated from the lower training ages of the Second League players. This determination is supported by the findings of the study conducted by Ergun et al. [18].

Considering the fact that wheelchair basketball is a sport branch that exerts high level of struggle effort, the player may need to practice for hours, days, or even months to improve himself/herself. As a result of the developments in training science, it is known that the quality of different training methods have been increased and therefore it is reflected in physical performance. It is possible to mention that the players with enhanced bio-motoric features can perform higher performances in the games.

## 5. Conclusions

In this study, it was considered important that anthropometric measurements of the wheelchair basketball players, modified bio-motoric test results, and field test findings particular to wheelchair basketball were all presented together. Moreover, it was a strong side of the study that the experimental group was composed of players from the highest two leagues among the wheelchair basketball leagues in Turkey. However, in order to reach more reliable results, it was considered more useful to conduct similar studies with a higher number of participants and according to other parameters. In conclusion, it was determined that wheelchair basketball players had similar anthropometric features in the First and Second League levels and that there was no difference based on the league level they were playing. Moreover, it was detected that bio-motor features and skills particular to wheelchair basketball were decisive on the levels of the leagues the players were playing. We evaluated that it was due to training ages. Besides, the difference between the leagues might be generated from the different competing levels among the wheelchair basketball leagues. It could be stated that the difference between the competing levels reflected on the physical profiles of the players.

## Figures and Tables

**Table 1:** The average values of anthropometric measurements of wheelchair basketball First League (1), and wheelchair basketball Second League (2), and Mann–Whitney U test results.

Group	*n*	Measurements			Parameters	Mann Whitney U		
		Min	Max	(Mean ± SD)		Mean Rank	U	*p*
1	12	1.00	4.00	2.75 ± 1.13	**Classification points**	11.17	52.000	0.880
2	9	1.00	4.00	2.83 ± 1.19		10.78		
1	12	19.00	48.00	34.33 ± 7.52	**Age (year)**	11.50	48.000	0.668
2	9	25.00	42.00	33.44 ± 5.70		10.33		
1	12	2.00	21.00	11.66 ± 6.75	**Training age (year)**	13.50	24.000	0.032 *
2	9	2.00	10.00	4.77 ± 2.27		7.67		
1	12	148.00	185.00	174.75 ± 9.36	**Height (cm)**	12.75	33.000	0.135
2	9	137.00	184.00	170.33 ± 13.08		8.67		
1	12	52.00	88.00	74.83 ± 11.73	**Weight (kg)**	11.29	50.500	0.803
2	9	47.00	86.00	75.22 ± 12.22		10.61		
1	12	17.53	29.75	24.53 ± 3.60	**BMI (kg/m^2^)**	10.00	42.000	0.394
2	9	21.86	28.73	25.80 ± 2.30		12.33		
1	12	42.50	65.00	55.77 ± 7.12	**Trunk length (cm)**	12.67	39.000	0.285
2	9	39.00	61.00	52.54 ± 6.39		9.75		
1	12	77.00	87.60	82.38 ± 3.51	**Upper extremity length (cm)**	14.13	16.500	0.057
2	9	72.00	81.00	78.11 ± 2.53		6.83		
1	12	29.50	37.50	32.56 ± 2.28	**Arm length (cm)**	13.92	19.000	0.062
2	9	28.50	32.50	30.33 ± 1.14		7.11		
1	12	26.00	31.00	28.48 ± 1.55	**Forearm length (cm)**	12.96	30.500	0.092
2	9	23.50	28.50	27.11 ± 1.51		8.39		
1	12	19.50	22.50	21.33 ± 0.86	**Hand length (cm)**	13.13	28.500	0.065
2	9	20.00	22.50	20.66 ± 0.82		8.17		
1	12	30.50	36.50	33.75 ± 1.80	**Arm circumference (cm)**	12.71	33.500	0.141
2	9	28.50	36.50	32.27 ± 2.39		8.72		
1	12	28.50	33.00	30.41 ± 1.56	**Forearm circumference (cm)**	14.08	17.000	0.078
2	9	25.50	31.00	28.05 ± 1.79		6.89		

* *p* < 0.05.

**Table 2:** The average values of performance measurements of wheelchair basketball First League (1), and wheelchair basketball Second League (2), and Mann–Whitney U test results.

Group	*n*	Measurements			Parameters	Mann Whitney U		
		Min	Max	(Mean ± SD)		Mean Rank	U	*p*
1	12	32.30	52.20	39.81 ± 6.51	**Trunk balance (cm)**	13.21	27.500	0.059
2	9	26.50	55.00	34.83 ± 8.71		8.06		
1	10	19.00	43.00	34.30 ± 6.60	**Modified sit-up (number)**	12.80	17.000	0.022 *
2	9	18.00	41.00	30.11 ± 5.84		6.89		
1	10	50.00	327.00	233.10 ± 78.04	**Modified abdominal endurance (s)**	13.30	12.000	0.007 *
2	9	34.00	225.00	112.44 ± 52.21		6.33		
1	10	24.00	40.00	33.20 ± 5.43	**Modified push up (number)**	10.45	40.500	0.712
2	9	26.00	41.00	32.55 ± 4.39		9.50		
1	12	45.00	77.00	62.00 ± 9.34	**Dominant hand grasping strength (kg)**	12.75	33.000	0.135
2	9	38.00	81.00	56.11 ± 12.06		8.67		
1	12	41.00	72.00	58.08 ± 9.52	**Non-dominant hand grasping strength (kg)**	12.63	34.500	0.164
2	9	36.00	79.00	53.11 ± 11.53		8.83		
1	12	7.34	12.18	9.45 ± 1.59	**Dominant hand plate tapping (s)**	8.42	23.000	0.028 *
2	9	9.13	16.80	11.74 ± 2.38		14.44		
1	12	8.78	13.03	11.07 ± 1.29	**Non-dominant hand plate tapping (s)**	8.83	28.000	0.065
2	9	10.44	17.60	13.33 ± 2.53		13.89		
1	12	−7.00	22.00	9.42 ± 9.85	**Right shoulder flexibility (cm)**	11.58	47.000	0.618
2	9	−7.00	21.00	7.83 ± 8.66		10.22		
1	12	−4.50	17.00	4.45 ± 8.02	**Left shoulder flexibility (cm)**	8.38	22.500	0.025 *
2	9	8.00	21.00	13.27 ± 4.03		14.50		
1	12	8.40	12.20	10.65 ± 1.02	**Pass for distance (m)**	13.38	25.500	0.043 *
2	9	6.00	12.50	9.04 ± 1.88		7.83		
1	12	5.16	6.60	5.75 ± 0.45	**20-m speed (s)**	8.63	25.500	0.043 *
2	9	5.45	6.90	6.20 ± 0.56		14.17		
1	12	11.03	13.69	12.15 ± 0.74	**Slalom without the ball (s)**	7.08	7.000	0.001 *
2	9	12.90	16.80	13.91 ± 1.12		16.22		
1	12	12.50	17.02	14.28 ± 1.25	**Slalom with the ball (s)**	8.08	19.000	0.013 *
2	9	14.76	25.00	16.89 ± 3.51		14.89		
1	12	19.00	26.00	23.58 ± 2.02	**Lay up (score)**	11.54	47.500	0.636
2	9	14.00	29.00	22.77 ± 4.14		10.28		
1	12	23.00	38.00	32.08 ± 4.33	**Zone shot (score)**	13.88	19.500	0.014 *
2	9	22.00	34.00	26.55 ± 3.97		7.17		
1	12	10.00	32.00	26.00 ± 5.89	**Pass for accuracy (score)**	13.71	21.500	0.020 *
2	9	3.00	30.00	19.66 ± 7.85		7.39		
1	12	1032.00	1311.00	1203.91 ± 99.94	**6-min endurance race (m)**	14.17	16.000	0.007 *
2	9	790.00	1140.00	1040.00 ± 112.9		6.78		

* *p* < 0.05.
